# Structure of racemic duloxetine hydro­chloride

**DOI:** 10.1107/S2056989023003353

**Published:** 2023-04-21

**Authors:** Mohan M. Bhadbhade, Jiabin Gao, Anne M. Rich, Christopher E. Marjo

**Affiliations:** a Mark Wainwright Analytical Centre, The University of New South Wales, UNSW, Sydney NSW 2052, Australia; b School of Chemistry, The University of New South Wales, UNSW Sydney NSW 2052, Australia; University of Aberdeen, United Kingdom

**Keywords:** anti-depressant drug, duloxetine, racemate, crystal structure, side chain conformation, mol­ecular packing

## Abstract

Duloxetine hydro­chloride (trade name Cymbalta) is marketed as a single enanti­omer (*S*)-*N*-methyl-3-(naphthalen-1-yl­oxy)-3-(thio­phen-2-yl)propyl­am­in­ium chloride, C_18_H_20_NOS^+^·Cl^−^, which is twice as effective as the (*R*)-enanti­omer in serotonin uptake. Here, we report the crystal structure of duloxetine hydro­chloride in its racemic form (space group *Pna*2_1_), where it shows significant differences in the mol­ecular conformation and packing in its extended structure compared to the previously reported (*S*)-enanti­omer crystal structure. Mol­ecules of this type, comprising aromatic groups with a single side chain terminated in a protonated secondary amine, are commonly found in active anti­depressants. A Cambridge Structural Database survey of mol­ecules with these features reveals a strong correlation between side-chain conformation and the crystal packing: an extended side chain leads to mol­ecules packed into separated layers of hydro­phobic and ionic hydro­philic phases. By comparison, mol­ecules with bent side chains, such as racemic duloxetine hydro­chloride, lead to crystal-packing motifs where an ionic hydro­philic phase is encapsulated within a hydro­phobic shell.

## Abstract 2

The structure of the hydro­chloride salt of the anti-depressant drug duloxetine in its racemic form shows differences in the side chain conformation and mol­ecular packing compared to its chirally pure crystal form. A Cambridge Database search of anti-depressant structures shows a high correlation between the side-chain conformation and the packing of mol­ecules, a feature of inter­est from a crystal engineering perspective.

## 1. Chemical context

Since 1957, major depressive disorder was mainly treated with tricyclic anti­depressants, a typical example being imipramine [(**1**) in scheme; brand name Tofranil; Azima & Vispo, 1958 ▸]. This family of tricyclic psychoactive drugs shares an aromatic or semi-aromatic tricyclic system and a flexible alkyl side chain of 2–3 carbon atoms with a terminal amine group. The primary mechanism of action is to block the reabsorption of the serotonin and norepinephrine neurotransmitters; however, other neurotransmitters were also blocked, leading to side effects including mouth dryness, blurred vision, and tachycardia. Increasing demand to treat mental disorders, such as depression and anxiety, has led to continuing efforts to develop new anti­depressants with more specific binding and fewer side effects. The newer family of drugs such as fluoxetine [(**2**) in scheme; Wong *et al.*, 1995 ▸; commercial name Prozac] came onto the market in 1986. This class of drugs act as selective serotonin reuptake inhibitors (SSRI) and are widely prescribed. Subsequent development resulted in the serotonin-norepinephrine reuptake inhibitor (SNRI) drug duloxetine [(**3**) is (*S*)-duloxetine hydrochloride in the scheme] that came onto the market in 2004. All anti­depressant drugs prior to duloxetine were formulated as racemates, whereas duloxetine was the first SNRI family drug sold as the chirally pure (*S*)-enanti­omer, as it was shown to be at least 2.5 times more active than the (*R*)-enanti­omer (Budău *et al.*, 2017 ▸). The recognition that a specific enanti­omer of an anti­depressant (as well as for many other classes of drugs) leads to more specific receptor binding and lesser side effects, has led to intensive research in this area by the pharmaceutical industry (Berton & Nestler, 2006 ▸; Brooks *et al.*, 2011 ▸). 
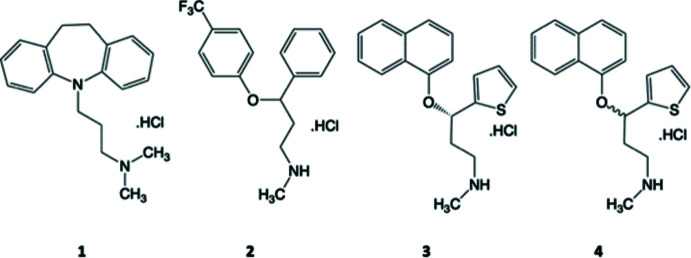


The crystal structure of duloxetine hydro­chloride can pro­vide valuable data on conformations and offers an experimentally observed starting point for receptor binding calculations, if the crystal structure of a drug–biomolecule complex cannot be obtained (Kitchen *et al.*, 2004 ▸). Earlier, the authors have reported the structure of the (*S*)-enanti­omer [(**3**); Bhadbhade *et al.*, 2009 ▸] and the acetone solvate of the (*S*)-enanti­omer, [(**3a**); Marjo *et al.*, 2011 ▸]. In view of the activity differences of the enanti­omers, the X-ray structure determination of the hydro­chloride salt of racemic duloxetine (**4**) was undertaken to examine the differences in mol­ecular conformation, inter­molecular inter­actions, and crystal packing in the respective crystals. The differences in the mol­ecular inter­actions lead to clear changes in the FTIR and Raman spectra between the racemic and enanti­opure crystal structures. This, to the best of our knowledge, is the first example of an SNRI drug to be characterized and compared both in its chirally pure and racemic forms. A Cambridge Crystallographic Data Centre (CCDC) survey of mol­ecular packing was carried out by searching for mol­ecules with side chains like those present in anti-depressants. A common packing motif was identified across all the examples in the survey that may be relevant to crystal-engineering studies.

## 2. Structural commentary

**Figure 1:**
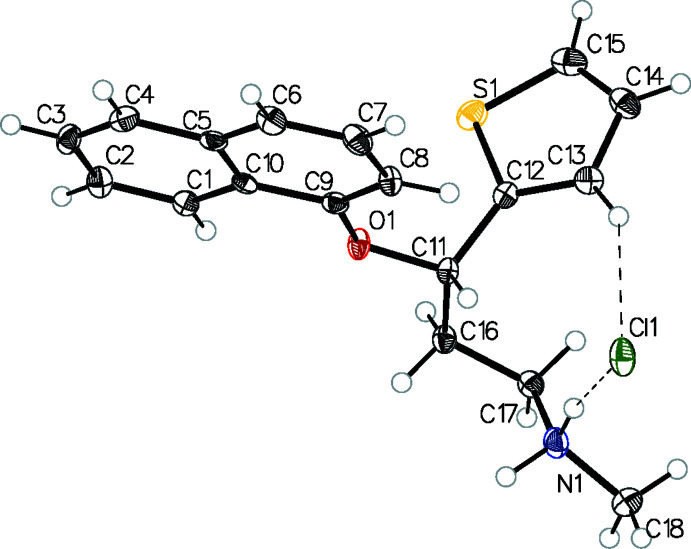
The mol­ecular structure of (**4**) with displacement ellipsoids drawn at the 50% probability level.

**Figure 2:**
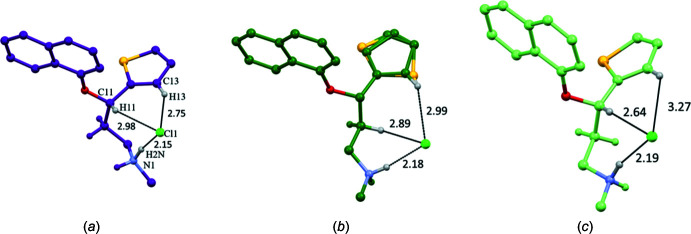
Views with displacement ellipsoids plotted at 50% probability level of the *S*-isomer of duloxetine in (*a*) (**4**) (purple), (*b*) (**3**) (dark green) and (*c*) (**3a**) (light green) in similar orientations. Significant contact distances (in Å) of the chloride ion with the cation are shown.

The chemical structure of (**4**) is shown in the scheme. The mol­ecular structure of (**4**), which crysallizes in space group *Pna*2_1_, is shown in Fig. 1 ▸: in the arbitrarily chosen asymmetric mol­ecule, atom C11 has an *S* configuration, but crystal symmetry generates a racemic mixture. A comparison of the *S*-enanti­omers of the duloxetinium cations in (**4**), (**3**) and (**3a**) are shown in Fig. 2 ▸. A noticeable difference is seen in the side-chain conformations; torsion angles in the side chain are similar in (**3**) and (**3a**) but quite different in (**4**): the major difference is in the first C—C torsion angle, which is 64.5 (3)° (*gauche*) in (**4**) compared to 168.0 (3)° in (**3**) and 164.3 (10)° in (**3a**) (both *anti*). The orientation of the protonated amino group towards the end of the side chain is also different in (**4**) compared to the other two structures. It is inter­esting that the fully extended side-chain conformation [as in (**3**) and (**3a**)] could be correlated with a distinct layering of the hydro­phobic and hydro­philic regions of the mol­ecules, which has a relevance in altering the solubility (and therefore bioavailability) of the formulation. This will be discussed in the database survey section. The Cl^−^ anion has different inter­actions with the duloxetinium cations in the three crystalline forms with different side-chain conformations. Although the N—H⋯Cl hydrogen bond is present in all three, as expected, other weaker inter­actions (C—H⋯Cl) show variations (Fig. 2 ▸). The C—H⋯Cl hydrogen bond in (**4**) formed by the thio­phene C13—H13 grouping is the shortest of the three structures. The thio­phene ring is orientationally disordered in (**3**) (Bhadbhade *et al.*, 2009 ▸), while in (**4**) the same ring is stabilized in a single orientation, probably because of the shorter C—H⋯Cl contact (2.75 Å) compared to the longer one (2.99 Å) in (**3**); the acetone mol­ecule makes C—H⋯π contacts to stabilize the orientation of the thio­phene ring in (**3a**). In the three crystalline forms (Fig. 2 ▸), the dihedral angle between the mean planes through the naphthalene ring system and thio­phene ring are similar in (**3**) and (**4**) [85.53 (6)° and 87.4 (6)°, respectively] and slightly smaller in (**3a**) [80..0 (4)°].

**Figure 3:**
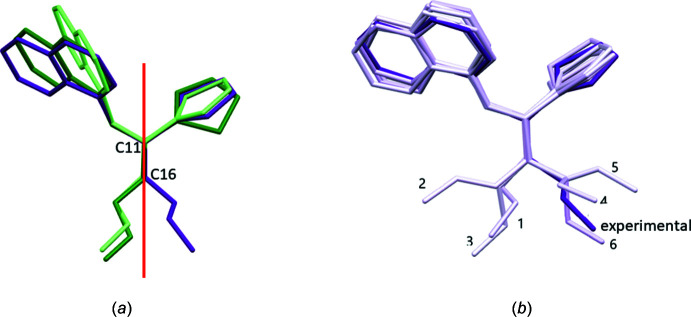
(*a*) Overlap of experimentally observed conformations of (**3**), (**3a**) and (**4**); (*b*) six best conformers generated for the *S*-form of the mol­ecule in (**4**) (light purple) using CSD-discovery (*Mercury*; Macrae *et al.*, 2020 ▸) – the dark purple one is the experimentally observed conformation.

The overlay of the three mol­ecules in (**3**), (**3a**) and (**4**) shows this side-chain conformational difference clearly (Fig. 3 ▸ *a*); the side chains are almost related by a virtual mirror perpend­icular to the plane of the paper (shown in red that passes through C11–C16). Inter­estingly, the first six conformers generated for the *S*-form of the mol­ecule in (**4**) using the *Mercury* software (Macrae *et al.*, 2020 ▸), show that both these conformations are likely to exist for a single mol­ecule. An overlap of the six computationally generated conformers (light purple) with the experimentally observed conformation (in dark purple) is shown in Fig. 3 ▸ *b*.

The differences in side-chain conformation also affect the Raman spectra where the methyl­ene scissoring mode at 1440 cm^−1^ for (**4**) splits into a doublet for (**3**). The peaks due to methyl­ene rocking between 700–750 cm^−1^ in the infrared are similar for (**3**) and (**3a**), but change in intensity for (**4**) (Fig. S3, supporting information).

## 3. Supra­molecular features

**Figure 4:**
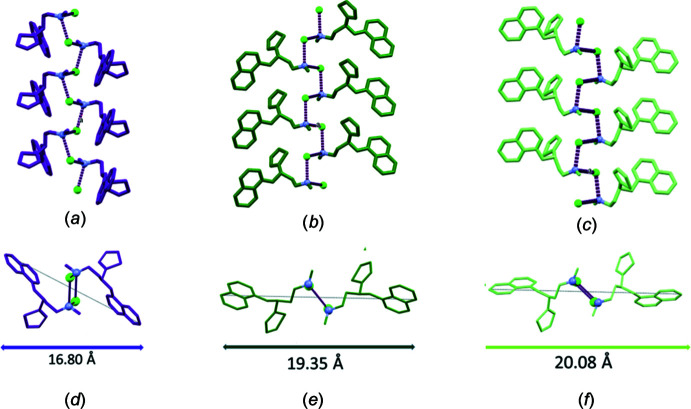
Two views of the helices generated by N—H⋯Cl hydrogen bonding across the 2_1_ screw axis in (**4**), (**3**) and (**3a**) looking perpendicular to [in (*a*), (*b*) and (*c*)] and down [in (*d*), (*e*) and (*f*)] the axis of the helix in the three structures, respectively.

The strong N—H⋯Cl hydrogen bonding inter­action in (**4**) (Table 1 ▸), which propagates along a crystallographic 2_1_ screw axis in all three crystals, as imposed by this symmetry, can only exist between the mol­ecules of the same chirality. Thus, in the crystal of (**4**), both the *S*- and *R*-enanti­omers form their own separate helices. In fact, this ‘chiral resolving’ feature of the N—H⋯Cl inter­action was exploited in achieving the resolution of a key inter­mediate of duloxetine with (*S*)-mandelic acid (Sakai *et al.*, 2003 ▸). However, in the case of (**4**), there was only a single crystalline phase, and we did not observe any conglomerates. A Rietveld analysis of the powder X-ray diffraction data of racemic duloxetine hydro­chloride demonstrated that the bulk crystalline material is the same phase as that found in the single-crystal measurements (Fig. S2, supporting information). A single helix in each of the three structures viewed perpendicular to and down the helix axis is shown in Fig. 4 ▸. As seen from the two views of the helix, the helix formed by (**3**) and (**3a**) are very similar, mol­ecules with their extended side chains forming an ‘opened up’ assembly, whereas the folded side chain is somewhat ‘closed’ in (**4**). The hydrogen-bonding parameters are given in Table 1 ▸.

**Figure 5:**
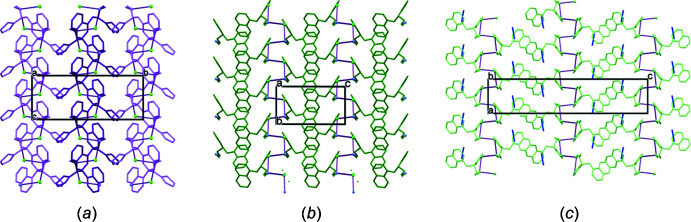
Differences in the packing of the N—H⋯Cl linked helices in structures of (*a*) (**4**) (the helix of the *S*-isomer is dark purple and that of the *R*-isomer is light purple), (*b*) (**3**) and (*c*) (**3a**) (acetone included is shown in blue). All are shown with the 2_1_ screw axis (marked) in the plane of the paper.

A striking difference in the helical chain packing in the three structures (Fig. 4 ▸) is the entanglement of aromatic groups. The naphthalene ring systems are inter­locked with the neighbouring helices in chiral structures (**3**) and (**3a**) (Fig. 4 ▸ *b* and *c*) whereas these large aromatic groups do not make inter-helical contacts in (**4**) (Fig. 4 ▸ *a*); rather, the thio­phene rings protrude to make inter­helical contacts. Comparison of inter­molecular inter­actions is shown in more detail only between structures (**3**) and (**4**), since (**3a**) contains an additional inter­acting solvent (acetone) mol­ecule (Fig. 5 ▸).

**Figure 6:**
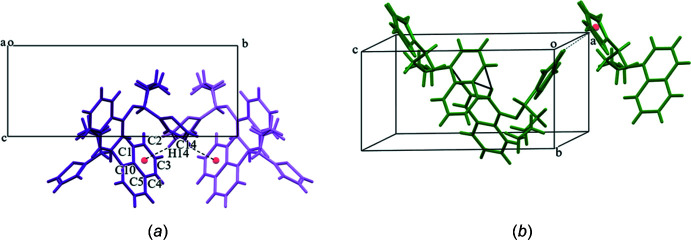
Difference in the C—H⋯π engagement of aromatic groups in (*a*) (**4**) and (*b*) (**3**) with mol­ecules viewed down the screw axis. The colour scheme is same as before; the *S*-isomer is dark purple and the *R*-isomer is light purple.

In (**4**), the thio­phene ring C14—H14 grouping makes C—H⋯π contacts with one of the rings of the naphthalene unit (C1–C5/C10) from the neighbouring helix as shown in Fig. 6 ▸ *a*, *i.e*., the *S*-isomer makes this contact with the *R*-isomer and *vice versa*. Conversely, in (**3**) (Fig. 6 ▸ *b*), the naphthalene groups inter­lock with each other (C7—H7 and C8—H8 make contacts with atoms C9 and C6, respectively) along the helix and the thio­phene rings make contacts with the neighbouring helix (C13*A*—H13*A* with the thio­phene ring). The locking of mol­ecules in the herringbone pattern of C—H⋯π contacts between the naphthalene groupings and the the adjacent thio­phene rings may explain the significantly higher melting point of (**3**) (441 K) compared to (**4**) (410 K). Although these inter­actions are not considered to be strong, this herringbone pattern of inter­locking mol­ecules is known to result in a higher melting point in aromatic systems with a similar V-shaped geometry (Gao *et al.*, 2017 ▸).

## 4. Database survey

A Cambridge Structural Database (CSD, Version 22.3.0; Groom *et al.*, 2016 ▸) search was carried out for common anti-depressants and mol­ecules similar to these drugs. The search allowed examination of any correlation between the side chain conformation and the mol­ecular organization in the solid state and how the duloxetine structures behave within this group. Mol­ecular packing is relevant in the design of co-crystals that can modulate the solubilities, and therefore bio-availabilities, of the formulations. Structural studies of co-crystals are indeed reported for fluoxetine (Childs *et al.*, 2004 ▸) and vortioxetine (Zhou *et al.*, 2016 ▸); however, there are no such studies of the co-crystals of duloxetine so far.

**Figure 7:**
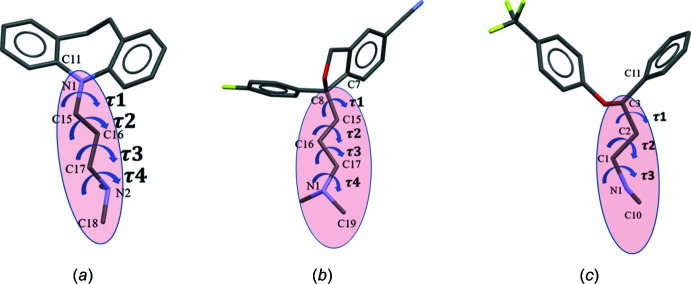
Cambridge Database search carried out only for the specific side chains (highlighted) that are present in three commonly prescribed anti-depressant mol­ecules (*a*) imipramine (*b*) citalopram and (*c*) fluoxetine. The torsion angles τ1, τ2, τ3 and τ4 describe the side-chain conformation.

**Figure 8:**
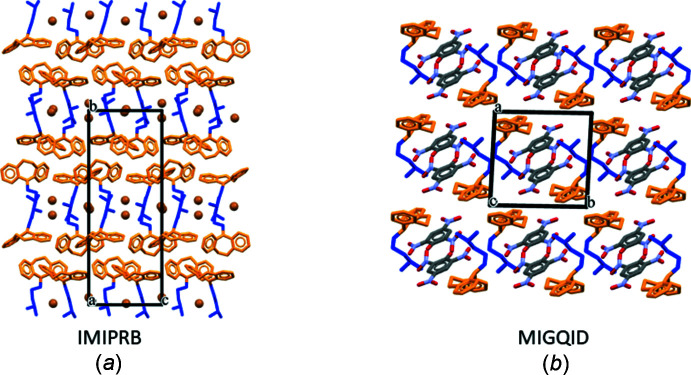
Selected examples of mol­ecular packing: (*a*) imipramine as its bromide salt (IMIPRB) with all four torsion angles close to ±180°, and (*b*) as its picrate salt, MIGQID, with τ3 deviating from 180°.

The CSD search was carried out on three mol­ecular classes differing in their side chains. The three classes corresponded to (i) those with an imipramine-type side chain, (ii) a citalopram-type side chain (linked to the aromatic residues *via* a carbon atom), and (iii) a fluoxetine-type that includes the duloxetine structures. Fig. 7 ▸ shows three anti­depressant mol­ecules; the highlighted side chain was used as a fragment in the CSD search using the program *CONQUEST* (Bruno *et al.*, 2002 ▸). A table of torsion angles is available in the deposited supporting information (S8); selected mol­ecular packing diagrams are shown in Fig. 8 ▸.

There were 28 structures for the imipramine-like class of which 9 contained the imipramine mol­ecular cation (including the chloro derivative) and 19 were analogues of imipramine. The number of hits for citalopram-like mol­ecules are fewer in number (8). The fluoxetine-like mol­ecules gave 21 hits and included the three duloxetine structures from this laboratory.

**Figure 9:**
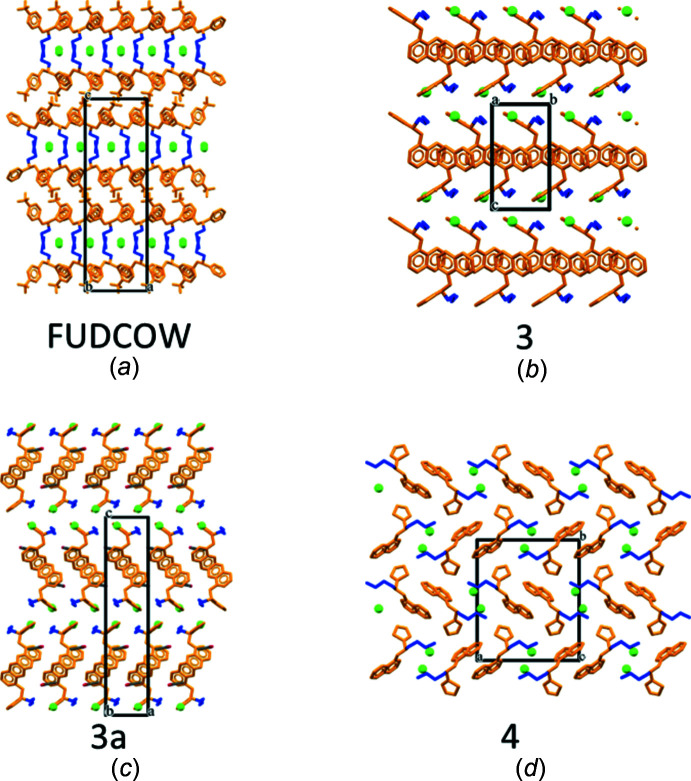
Selected examples of mol­ecules with shorter side chains: (*a*) fluoxetine with all three side-chain torsion angles ∼180°; packing in three crystalline environments of duloxetine; (*b*) chirally pure *S*-isomer, (**3**), (*c*) the acetone solvate of the *S*-isomer, (**3a**) and (*d*) racemic duloxetine, (**4**).

Three tables of torsion angles (S8) and the mol­ecular packing diagrams viewed down suitable directions (S9) are included in the supporting information. Representative packing diagrams (Fig. 8 ▸ *a*) show the aromatic groups clustered together and alkyl amino chains and anions separated from the hydro­phobic aromatic groups. When the τ3 torsion angle deviates significantly from 180°, corresponding to a bend at the chain terminus, the hydro­phobic–hydro­philic segregation no longer occurs (Fig. 8 ▸ *b*). Although there are fewer structures in the citalopram category, all have the four torsion angles close to ±180°, indicating a fully extended side chain. As observed in mol­ecules with an imipramine-like side chain, these mol­ecules pack with their aromatic and alkyl amino chains forming distinct layers and representative examples are shown in Fig. 9 ▸.

Analysis of all three groups shows a strong correlation in mol­ecular packing with the side chain conformation. A fully extended side chain leads to packing of mol­ecules that have distinctly separated layers: a hydro­phobic layer where the aromatic residues engage in C—H⋯π and π–π inter­actions and a hydro­philic layer of alkyl­amino side chains bearing a positively charged nitro­gen atom and anion. By comparison, bent or folded side chains do not show such a marked distinction. We define a layered structure here as one where the aromatic groups assemble *via* classical inter­molecular inter­actions (C—H⋯π, π–π) into sheets extending infinitely in two dimensions. These aromatic sheets are separated by a charged layer of aliphatic chains and anions. Non-layered structures are classified by those where discrete ionic hydro­philic regions are encapsulated by a hydro­phobic shell.

The last category, with mol­ecules having a shorter fluoxetine-like chain, follow a similar pattern. The first 10 entries have τ1, τ2 and τ3 torsion angles close to ±180° leading to clustering of the aromatic groups into layers separating the hydro­philic side chains and anions (Fig. 9 ▸ *a*). In the cases where τ2 or τ3 deviates significantly from ±180° (entries 11–18), this has not made a significant difference to the mol­ecular organization and the separation still seems to take place. Presumably this is due to reduced demand for space from the shorter chain in these mol­ecules.

It is inter­esting that the structures of duloxetine do not follow this pattern of mol­ecular association observed in the previous three classes (Fig. 9 ▸ *b*, *c* and *d*). The *S*-form (**3**) does show the layered structure with naphthalene groups engaged in C—H⋯π contacts forming a layer, but the next layer has an alkyl­amino chain and the thio­phene ring as well in it (Fig. 9 ▸ *b*). Similar packing is observed in the *S*-form of the acetone solvate (**3a**) (Fig. 9 ▸ *c*) and the structure of the racemate does not show any type of layering, with mol­ecules interacting discretely with each other without any clustering of the naphthalene groups observed in chiral structures (Fig. 9 ▸ *d*).

The analysis of mol­ecular packing from the CSD search points to three factors responsible in the mol­ecular organization: (i) mol­ecules with a fully extended side chain (all side-chain torsion angles approach 180°) with small organic anions or halide ions form well-separated layers; (ii) large aromatic organic anions inter­act and disrupt aromatic clustering of the aromatic groups in the mol­ecule (see, for example, Figure S9 in the supporting information, REZBII and ZUWHOO); (iii) larger aromatics, like naphthalene, tend to self-inter­act causing the aromatic heterocyclics to protrude into the hydro­philic layer, as in the (**3**) and (**3a**) forms and *S*-acetone.

Co-crystal formation utilizing the amine hydro­chlorides has been carried out to explore the improvement in active pharmaceutical ingredients (APIs) (Childs *et al.*, 2004 ▸). Duloxetine has a desirable profile as a next-generation anti-depressant, yet no studies on varying co-crystals with this mol­ecule have emerged. The CSD analysis presented above provides a valuable crystal-engineering approach for undertaking such investigations with a view to improving the biological efficacy of this pharmaceutical.

## 5. Synthesis and crystallization

Duloxetine hydro­chloride as the (*S*)-enanti­omer (**3**), and the racemate (**4**), were supplied by Arrow Pharmaceuticals as fine microcrystalline powders. Compound (**4**) was crystallized from its anhydrous ethanol solution by slow evaporation to produce thin long plates. The crystal structures of (**3**) and (**3a**) have been reported previously (Bhadbhade *et al.*, 2009 ▸; Marjo *et al.*, 2011 ▸).

Differential scanning calorimetry (DSC): the melting points of (**3**) and (**4**) were measured using a TA Instruments 2010 DSC V4.4E in an aluminium pan under a nitro­gen atmosphere with a heating rate of 5°C min^−1^ (Fig. S1, supporting information). Crystals of (**3a**) were very unstable and unsuitable for DSC measurements.

FTIR spectroscopy was performed using a PerkinElmer Spectrum 100 FTIR spectrometer fitted with a deuterated triglycine sulfate (dTGS) detector. Spectra were recorded as pure crystalline powders applied using a piston against a diamond/ZnSe ATR crystal. Each spectrum was an average of 64 scans with a spectral resolution of 4 cm^−1^.

Raman spectroscopy was performed using a PerkinElmer Raman Station 400F spectrometer, equipped with a high-resolution echelle spectrograph and a 785 nm excitation source (∼100 mW at the sample). 64 scans were acquired, and the baseline corrected for fluorescence.

Powder X-ray diffraction (PXRD): A sample of (**4**) (1g) was dissolved with slight warming in anhydrous ethanol (10 ml) and the solution allowed to evaporate under a stream of nitro­gen over several hours in a foil-covered beaker. The resulting oil was left to stand overnight to produce a crystalline mass that was collected and packed into a 20 mm stainless steel sample holder and X-ray diffraction peaks between 5° and 80° of 2θ measured using Cu *K*α radiation on a MPD X-ray diffractometer (PANalytical, Netherlands) fitted with a Pixel array detector. A Rietveld analysis of the data was performed using the single-crystal data CIF file of (**4**) to sim­ulate the powder diffraction pattern using the *High Score Plus* package (PANalytical, Netherlands). The refinement included an allowance for preferred orientation of the crystallites, as well as some refinement of the lattice dimensions, resulting in a weighted profile *R*-factor of 14.69%.

## 6. Refinement

Crystal data, data collection and structure refinement details are summarized in Table 2 ▸. All non-H atoms were treated anisotropically and H atoms (fixed at stereochemically reasonable positions) were kept isotropic.

## Supplementary Material

Crystal structure: contains datablock(s) I, global. DOI: 10.1107/S2056989023003353/hb8060sup1.cif

Structure factors: contains datablock(s) I. DOI: 10.1107/S2056989023003353/hb8060Isup2.hkl

Supporting information file. DOI: 10.1107/S2056989023003353/hb8060Isup4.cml

supporting information. DOI: 10.1107/S2056989023003353/hb8060sup3.pdf

CCDC reference: 2033424

Additional supporting information:  crystallographic information; 3D view; checkCIF report

## Figures and Tables

**Table 1:** Hydrogen-bond geometry (Å, °)

*D*—H⋯*A*	*D*—H	H⋯*A*	*D*⋯*A*	*D*—H⋯*A*
N1—H1*N*⋯Cl1^i^	0.97	2.23	3.127 (2)	154
N1—H2*N*⋯Cl1	0.97	2.15	3.106 (2)	168
C13—H13⋯Cl1	0.93	2.75	3.590 (3)	151
C15—H15⋯Cl1^ii^	0.93	2.86	3.685 (3)	149
C18—H181⋯Cl1^iii^	0.96	2.83	3.634 (3)	142
C18—H183⋯S1^iv^	0.96	2.98	3.829 (3)	148

Symmetry codes: (i) 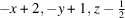 ; (ii) 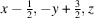 ; (iii) 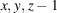 ; (iv) 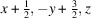 .

**Table 2:** Experimental details

Crystal data	
Chemical formula	C_18_H_20_NOS^+^·Cl^−^
*M*_r_	333.86
Crystal system, space group	Orthorhombic, *P* *n* *a*2_1_
Temperature (K)	100
*a*, *b*, *c* (Å)	14.587 (3), 17.250 (3), 6.8040 (14)
*V* (Å^3^)	1712.1 (6)
*Z*	4
Radiation type	Synchrotron, λ = 0.71073 Å
μ (mm^−1^)	0.35
Crystal size (mm)	0.04 × 0.02 × 0.01

Data collection	
Diffractometer	3-BM1 Australian Synchrotron
No. of measured, independent and observed [*I* > 2σ(*I*)] reflections	18750, 2573, 2546
*R*_int_	0.022
θ_max_ (°)	23.9
(sin θ/λ)_max_ (Å^−1^)	0.569

Refinement	
*R*[*F* ^2^ > 2σ(*F* ^2^)], *wR*(*F* ^2^), *S*	0.025, 0.066, 1.10
No. of reflections	2573
No. of parameters	200
No. of restraints	1
H-atom treatment	H-atom parameters constrained
Δρ_max_, Δρ_min_ (e Å^−3^)	0.13, −0.31
Absolute structure	Flack *x* determined using 1143 quotients [(*I* ^+^)−(*I* ^−^)]/[(*I* ^+^)+(*I* ^−^)] (Parsons *et al.*, 2013 ▸)
Absolute structure parameter	0.048 (12)

Computer programs: *BLU-ICE* (McPhillips *et al.*, 2002 ▸), *XDS* (Kabsch, 1993 ▸), *SHELXS97* (Sheldrick, 2008 ▸), *SHELXL2013* (Sheldrick, 2015 ▸) and *Mercury* (Macrae *et al.*, 2020 ▸).

## supplementary crystallographic information

### Crystal data

**Table:**

C_18_H_20_NOS^+^·Cl^−^	*D*_x_ = 1.295 Mg m^−^^3^
*M**_r_* = 333.86	Synchrotron radiation, λ = 0.71073 Å
Orthorhombic, *P**n**a*2_1_	Cell parameters from 9980 reflections
*a* = 14.587 (3) Å	θ = 2.5–22.5°
*b* = 17.250 (3) Å	µ = 0.35 mm^−^^1^
*c* = 6.8040 (14) Å	*T* = 100 K
*V* = 1712.1 (6) Å^3^	Thin plates, colourless
*Z* = 4	0.04 × 0.02 × 0.01 mm
*F*(000) = 704	

### Data collection

**Table:**

3-BM1 Australian Synchrotron diffractometer	2546 reflections with *I* > 2σ(*I*)
Radiation source: Synchrotron BM	*R*_int_ = 0.022
Si<111> monochromator	θ_max_ = 23.9°, θ_min_ = 2.7°
φ scans	*h* = −16→16
18750 measured reflections	*k* = −19→19
2573 independent reflections	*l* = −7→7

### Refinement

**Table:**

Refinement on *F*^2^	Hydrogen site location: inferred from neighbouring sites
Least-squares matrix: full	H-atom parameters constrained
*R*[*F*^2^ > 2σ(*F*^2^)] = 0.025	*w* = 1/[σ^2^(*F*_o_^2^) + (0.0442*P*)^2^ + 0.4827*P*] where *P* = (*F*_o_^2^ + 2*F*_c_^2^)/3
*wR*(*F*^2^) = 0.066	(Δ/σ)_max_ < 0.001
*S* = 1.10	Δρ_max_ = 0.13 e Å^−^^3^
2573 reflections	Δρ_min_ = −0.31 e Å^−^^3^
200 parameters	Absolute structure: Flack *x* determined using 1143 quotients [(*I*^+^)-(*I*^-^)]/[(*I*^+^)+(*I*^-^)] (Parsons *et al.*, 2013)
1 restraint	Absolute structure parameter: 0.048 (12)
Primary atom site location: structure-invariant direct methods	

### Special details

**Table:**

Geometry. All esds (except the esd in the dihedral angle between two l.s. planes) are estimated using the full covariance matrix. The cell esds are taken into account individually in the estimation of esds in distances, angles and torsion angles; correlations between esds in cell parameters are only used when they are defined by crystal symmetry. An approximate (isotropic) treatment of cell esds is used for estimating esds involving l.s. planes.

### Fractional atomic coordinates and isotropic or equivalent isotropic displacement parameters (Å^2^)

**Table:**

	*x*	*y*	*z*	*U*_iso_*/*U*_eq_	
S1	0.68113 (4)	0.71438 (4)	0.79051 (11)	0.02191 (19)	
O1	0.71913 (12)	0.54577 (10)	0.6675 (3)	0.0164 (4)	
N1	0.99472 (15)	0.60728 (13)	0.4867 (3)	0.0195 (5)	
H2N	0.9985	0.5990	0.6275	0.023*	
H1N	0.9870	0.5571	0.4246	0.023*	
C1	0.56705 (17)	0.45396 (14)	0.6123 (4)	0.0151 (5)	
H1	0.5858	0.4864	0.5109	0.018*	
C2	0.49137 (19)	0.40762 (15)	0.5880 (4)	0.0187 (6)	
H2	0.4598	0.4082	0.4692	0.022*	
C3	0.46130 (18)	0.35931 (15)	0.7416 (4)	0.0201 (6)	
H3	0.4096	0.3285	0.7245	0.024*	
C4	0.50779 (17)	0.35750 (14)	0.9157 (5)	0.0187 (6)	
H4	0.4868	0.3256	1.0163	0.022*	
C5	0.58737 (16)	0.40314 (13)	0.9468 (4)	0.0154 (5)	
C6	0.63645 (19)	0.40149 (15)	1.1258 (4)	0.0199 (6)	
H6	0.6181	0.3682	1.2258	0.024*	
C7	0.7104 (2)	0.44843 (16)	1.1528 (4)	0.0211 (6)	
H7	0.7417	0.4473	1.2720	0.025*	
C8	0.74062 (18)	0.49916 (15)	1.0014 (4)	0.0167 (5)	
H8	0.7908	0.5314	1.0223	0.020*	
C9	0.69579 (17)	0.50056 (14)	0.8253 (4)	0.0150 (5)	
C10	0.61698 (16)	0.45263 (13)	0.7919 (4)	0.0129 (5)	
C11	0.80007 (17)	0.59323 (14)	0.6816 (4)	0.0136 (5)	
H11	0.8499	0.5628	0.7395	0.016*	
C12	0.78314 (16)	0.66411 (13)	0.8064 (4)	0.0141 (5)	
C13	0.84199 (18)	0.69974 (14)	0.9345 (4)	0.0173 (5)	
H13	0.9006	0.6816	0.9620	0.021*	
C14	0.8036 (2)	0.76766 (16)	1.0212 (4)	0.0217 (6)	
H14	0.8344	0.7985	1.1118	0.026*	
C15	0.7175 (2)	0.78229 (15)	0.9574 (5)	0.0232 (6)	
H15	0.6822	0.8241	0.9990	0.028*	
C16	0.82355 (17)	0.61227 (14)	0.4678 (4)	0.0165 (6)	
H161	0.8271	0.5643	0.3938	0.020*	
H162	0.7742	0.6430	0.4122	0.020*	
C17	0.91328 (16)	0.65628 (13)	0.4429 (4)	0.0162 (5)	
H171	0.9176	0.6752	0.3089	0.019*	
H172	0.9133	0.7008	0.5298	0.019*	
C18	1.08132 (18)	0.64242 (16)	0.4174 (5)	0.0247 (6)	
H183	1.0898	0.6918	0.4797	0.037*	
H181	1.0788	0.6493	0.2775	0.037*	
H182	1.1317	0.6090	0.4501	0.037*	
Cl1	1.03494 (4)	0.57061 (3)	0.92525 (11)	0.02290 (19)	

### Atomic displacement parameters (Å^2^)

**Table:**

	*U*^11^	*U*^22^	*U*^33^	*U*^12^	*U*^13^	*U*^23^
S1	0.0157 (3)	0.0237 (3)	0.0264 (4)	0.0031 (3)	−0.0013 (3)	0.0015 (3)
O1	0.0184 (9)	0.0167 (9)	0.0139 (10)	−0.0067 (7)	−0.0032 (7)	0.0038 (7)
N1	0.0194 (11)	0.0141 (11)	0.0249 (14)	−0.0019 (8)	−0.0022 (9)	−0.0016 (9)
C1	0.0157 (12)	0.0097 (12)	0.0198 (15)	0.0033 (10)	−0.0006 (11)	0.0011 (10)
C2	0.0182 (14)	0.0154 (13)	0.0223 (15)	0.0007 (11)	−0.0053 (11)	0.0010 (11)
C3	0.0149 (13)	0.0126 (13)	0.0330 (18)	−0.0011 (10)	0.0002 (11)	−0.0004 (11)
C4	0.0190 (12)	0.0117 (12)	0.0255 (15)	0.0012 (9)	0.0067 (12)	0.0034 (12)
C5	0.0172 (12)	0.0107 (10)	0.0182 (14)	0.0040 (9)	0.0051 (11)	−0.0018 (11)
C6	0.0297 (15)	0.0131 (13)	0.0168 (15)	−0.0030 (11)	0.0051 (11)	0.0029 (10)
C7	0.0293 (15)	0.0187 (14)	0.0153 (16)	−0.0005 (12)	−0.0018 (12)	−0.0002 (11)
C8	0.0183 (12)	0.0143 (12)	0.0175 (13)	−0.0033 (10)	−0.0024 (11)	0.0003 (10)
C9	0.0168 (12)	0.0117 (11)	0.0165 (14)	0.0021 (10)	0.0037 (11)	−0.0010 (11)
C10	0.0152 (12)	0.0082 (10)	0.0154 (13)	0.0045 (9)	0.0007 (11)	−0.0017 (10)
C11	0.0135 (12)	0.0128 (12)	0.0145 (13)	−0.0023 (10)	−0.0017 (10)	0.0012 (11)
C12	0.0142 (11)	0.0145 (11)	0.0137 (14)	−0.0003 (10)	−0.0009 (11)	0.0035 (11)
C13	0.0181 (12)	0.0182 (12)	0.0156 (13)	0.0029 (10)	−0.0018 (11)	0.0028 (11)
C14	0.0324 (15)	0.0155 (13)	0.0172 (14)	−0.0016 (12)	0.0019 (12)	0.0002 (11)
C15	0.0307 (15)	0.0153 (12)	0.0237 (16)	0.0036 (10)	0.0082 (13)	0.0032 (12)
C16	0.0167 (13)	0.0178 (12)	0.0149 (15)	−0.0028 (9)	−0.0023 (10)	0.0004 (11)
C17	0.0189 (12)	0.0139 (11)	0.0158 (14)	−0.0005 (9)	0.0001 (11)	0.0007 (12)
C18	0.0172 (13)	0.0286 (14)	0.0283 (16)	0.0002 (10)	0.0018 (13)	−0.0045 (14)
Cl1	0.0234 (3)	0.0141 (3)	0.0311 (4)	−0.0013 (2)	−0.0104 (3)	0.0026 (3)

### Geometric parameters (Å, º)

**Table:**

S1—C15	1.715 (3)	C7—H7	0.9300
S1—C12	1.726 (2)	C8—C9	1.365 (4)
O1—C9	1.370 (3)	C8—H8	0.9300
O1—C11	1.440 (3)	C9—C10	1.434 (3)
N1—C18	1.478 (4)	C11—C12	1.509 (4)
N1—C17	1.488 (3)	C11—C16	1.531 (4)
N1—H2N	0.9700	C11—H11	0.9800
N1—H1N	0.9700	C12—C13	1.369 (4)
C1—C2	1.373 (4)	C13—C14	1.426 (4)
C1—C10	1.423 (4)	C13—H13	0.9300
C1—H1	0.9300	C14—C15	1.353 (4)
C2—C3	1.407 (4)	C14—H14	0.9300
C2—H2	0.9300	C15—H15	0.9300
C3—C4	1.365 (4)	C16—C17	1.523 (3)
C3—H3	0.9300	C16—H161	0.9700
C4—C5	1.419 (4)	C16—H162	0.9700
C4—H4	0.9300	C17—H171	0.9700
C5—C6	1.413 (4)	C17—H172	0.9700
C5—C10	1.423 (4)	C18—H183	0.9600
C6—C7	1.362 (4)	C18—H181	0.9600
C6—H6	0.9300	C18—H182	0.9600
C7—C8	1.422 (4)		
			
C15—S1—C12	92.03 (13)	C1—C10—C9	122.5 (2)
C9—O1—C11	118.4 (2)	O1—C11—C12	111.4 (2)
C18—N1—C17	112.7 (2)	O1—C11—C16	104.0 (2)
C18—N1—H2N	109.1	C12—C11—C16	113.4 (2)
C17—N1—H2N	109.1	O1—C11—H11	109.3
C18—N1—H1N	109.1	C12—C11—H11	109.3
C17—N1—H1N	109.1	C16—C11—H11	109.3
H2N—N1—H1N	107.8	C13—C12—C11	128.3 (2)
C2—C1—C10	120.4 (2)	C13—C12—S1	110.78 (19)
C2—C1—H1	119.8	C11—C12—S1	120.89 (18)
C10—C1—H1	119.8	C12—C13—C14	112.7 (2)
C1—C2—C3	120.4 (3)	C12—C13—H13	123.6
C1—C2—H2	119.8	C14—C13—H13	123.6
C3—C2—H2	119.8	C15—C14—C13	112.6 (3)
C4—C3—C2	120.2 (2)	C15—C14—H14	123.7
C4—C3—H3	119.9	C13—C14—H14	123.7
C2—C3—H3	119.9	C14—C15—S1	111.8 (2)
C3—C4—C5	121.6 (3)	C14—C15—H15	124.1
C3—C4—H4	119.2	S1—C15—H15	124.1
C5—C4—H4	119.2	C17—C16—C11	113.9 (2)
C6—C5—C4	122.1 (2)	C17—C16—H161	108.8
C6—C5—C10	119.8 (2)	C11—C16—H161	108.8
C4—C5—C10	118.1 (2)	C17—C16—H162	108.8
C7—C6—C5	120.4 (2)	C11—C16—H162	108.8
C7—C6—H6	119.8	H161—C16—H162	107.7
C5—C6—H6	119.8	N1—C17—C16	112.38 (19)
C6—C7—C8	120.9 (3)	N1—C17—H171	109.1
C6—C7—H7	119.5	C16—C17—H171	109.1
C8—C7—H7	119.5	N1—C17—H172	109.1
C9—C8—C7	119.9 (2)	C16—C17—H172	109.1
C9—C8—H8	120.0	H171—C17—H172	107.9
C7—C8—H8	120.0	N1—C18—H183	109.5
C8—C9—O1	125.4 (2)	N1—C18—H181	109.5
C8—C9—C10	120.9 (2)	H183—C18—H181	109.5
O1—C9—C10	113.8 (2)	N1—C18—H182	109.5
C5—C10—C1	119.4 (2)	H183—C18—H182	109.5
C5—C10—C9	118.1 (2)	H181—C18—H182	109.5
			
C10—C1—C2—C3	1.2 (4)	O1—C9—C10—C5	179.7 (2)
C1—C2—C3—C4	−0.7 (4)	C8—C9—C10—C1	178.3 (2)
C2—C3—C4—C5	−0.5 (4)	O1—C9—C10—C1	−1.4 (3)
C3—C4—C5—C6	−179.6 (2)	C9—O1—C11—C12	−76.4 (3)
C3—C4—C5—C10	1.2 (3)	C9—O1—C11—C16	161.1 (2)
C4—C5—C6—C7	−177.4 (2)	O1—C11—C12—C13	143.3 (3)
C10—C5—C6—C7	1.7 (4)	C16—C11—C12—C13	−99.8 (3)
C5—C6—C7—C8	−0.8 (4)	O1—C11—C12—S1	−40.0 (3)
C6—C7—C8—C9	−0.7 (4)	C16—C11—C12—S1	76.9 (3)
C7—C8—C9—O1	−178.9 (2)	C15—S1—C12—C13	−0.6 (2)
C7—C8—C9—C10	1.4 (4)	C15—S1—C12—C11	−177.8 (2)
C11—O1—C9—C8	3.4 (3)	C11—C12—C13—C14	177.5 (2)
C11—O1—C9—C10	−176.9 (2)	S1—C12—C13—C14	0.5 (3)
C6—C5—C10—C1	−179.9 (2)	C12—C13—C14—C15	−0.2 (4)
C4—C5—C10—C1	−0.8 (3)	C13—C14—C15—S1	−0.2 (3)
C6—C5—C10—C9	−1.0 (3)	C12—S1—C15—C14	0.4 (2)
C4—C5—C10—C9	178.1 (2)	O1—C11—C16—C17	−174.42 (19)
C2—C1—C10—C5	−0.4 (3)	C12—C11—C16—C17	64.5 (3)
C2—C1—C10—C9	−179.3 (2)	C18—N1—C17—C16	166.0 (2)
C8—C9—C10—C5	−0.5 (3)	C11—C16—C17—N1	70.1 (3)

### Hydrogen-bond geometry (Å, º)

**Table:**

*D*—H···*A*	*D*—H	H···*A*	*D*···*A*	*D*—H···*A*
N1—H1*N*···Cl1^i^	0.97	2.23	3.127 (2)	154
N1—H2*N*···Cl1	0.97	2.15	3.106 (2)	168
C13—H13···Cl1	0.93	2.75	3.590 (3)	151
C15—H15···Cl1^ii^	0.93	2.86	3.685 (3)	149
C18—H181···Cl1^iii^	0.96	2.83	3.634 (3)	142
C18—H183···S1^iv^	0.96	2.98	3.829 (3)	148

Symmetry codes: (i) −*x*+2, −*y*+1, *z*−1/2; (ii) *x*−1/2, −*y*+3/2, *z*; (iii) *x*, *y*, *z*−1; (iv) *x*+1/2, −*y*+3/2, *z*.
