# Supplementary material for: Structure of racemic duloxetine hydro­chloride
Source: Acta Crystallogr E Crystallogr Commun. 2023 Apr 21;79(Pt 5):488–93. doi: 10.1107/S2056989023003353 (PMC10162076; doi:10.1107/S2056989023003353)

STRUCTURE REPORTS

Volume 79 (2023)

Supporting information for article:

Structure of racemic duloxetine hydrochloride

Mohan M. Bhadbhade, Jiabin Gao, Anne M. Rich and Christopher E. Marjo

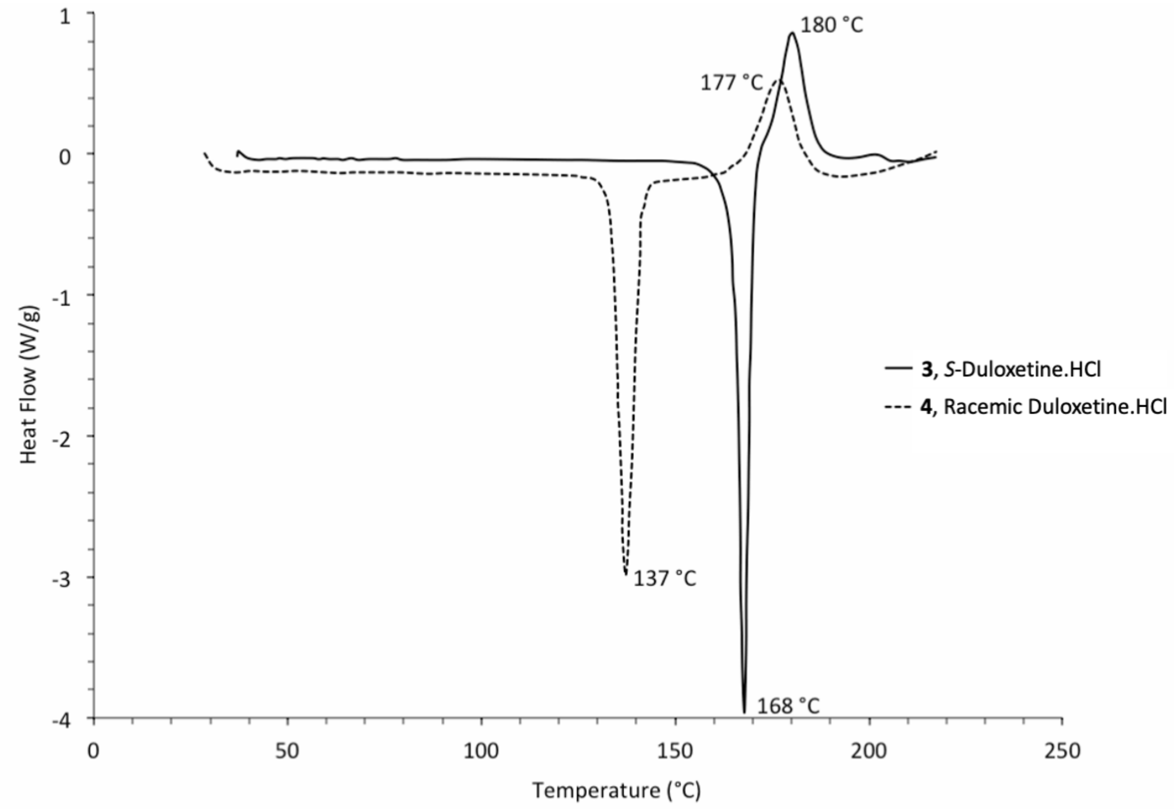

Fig. S1: DSC runs of *S*-duloxetine, **3**, and duloxetine racemate, **4**.

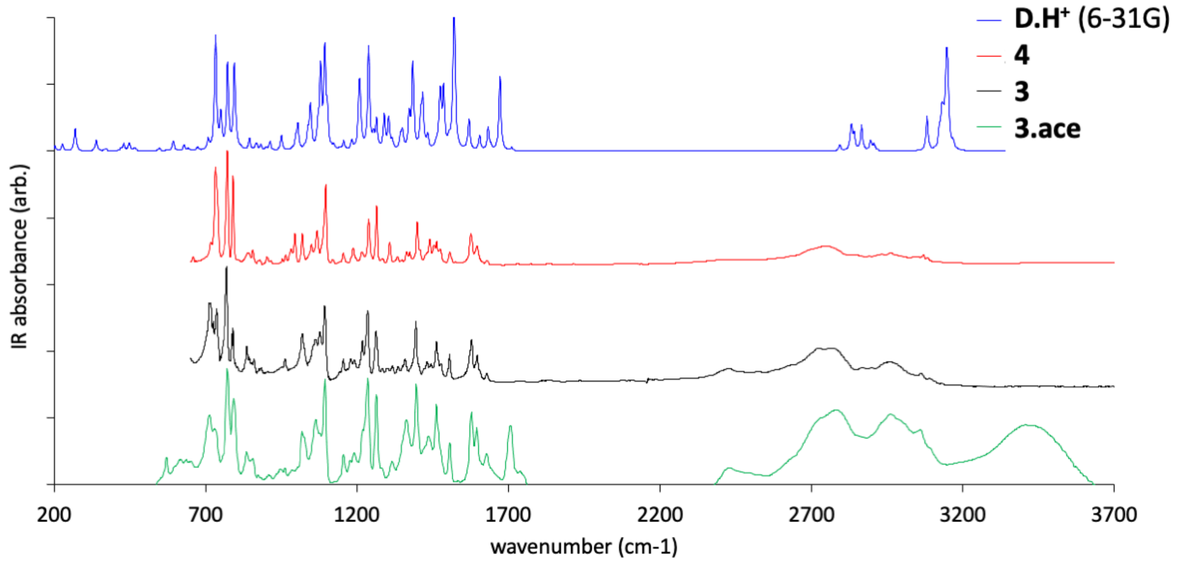

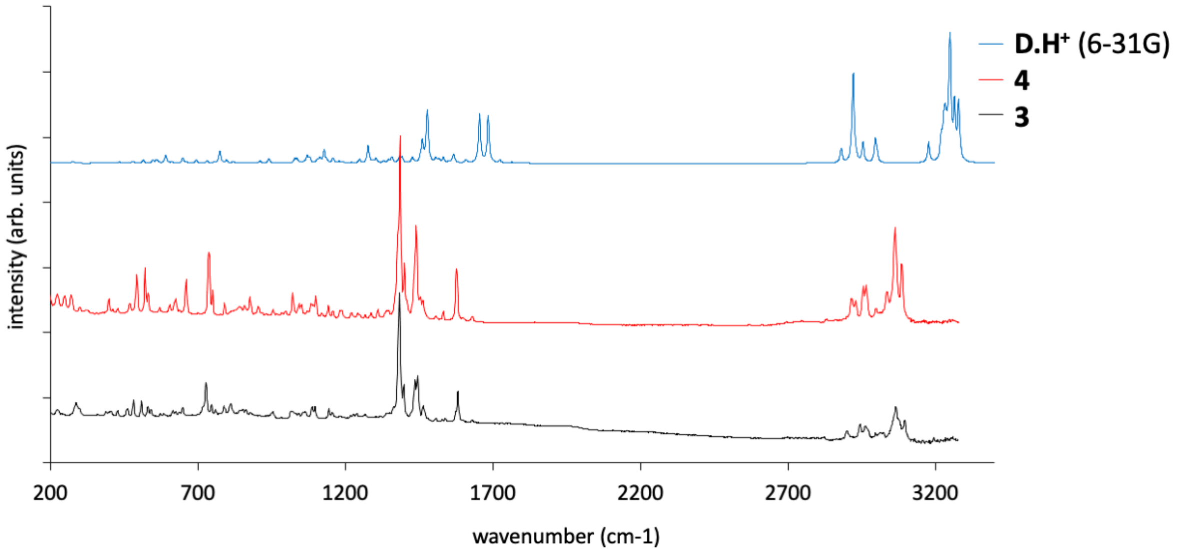

Figure S2: Upper stack plot, FTIR spectra between 650-3700 cm^{-1} calculated for gas state of ***duloxetine.H^{+}*** *(DFT 6-31G basis set), and measured for* ***4***, ***3*** *and* ***3.ace***; *Lower stack plot, Raman spectra between (200-3250 cm^{-1}) of gas state of* ***duloxetine.H^{+}*** *(DFT 6-31G basis set), and measured spectra of* ***4*** *and* ***3***.

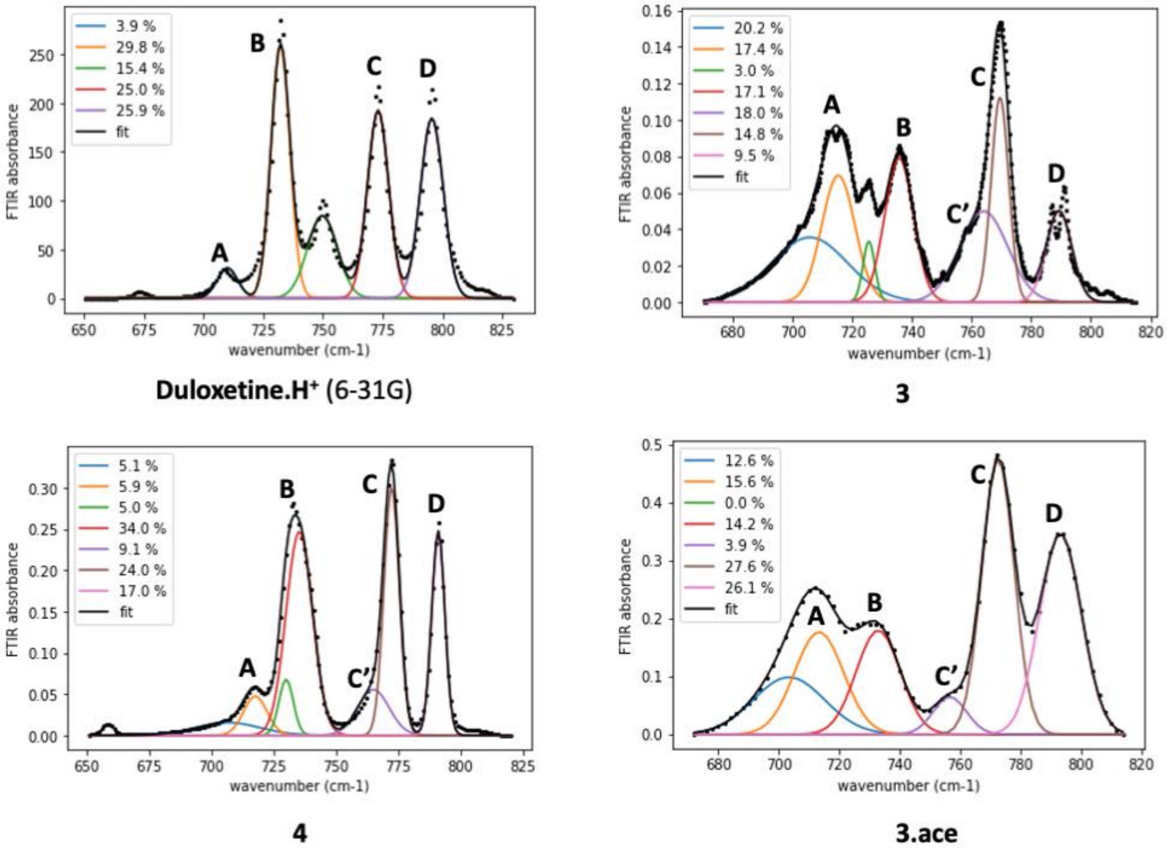

*Figure S3: Gaussian fits to FTIR spectra from 670-810 cm^{-1} for gas state **duloxetine.H**$^{+}$ (6-31G), **3**, **4** and **3.ace**. Peak area ratios were calculated from the area under peak A (orange) and the combination of peaks C' and C (purple and brown).*

Table S4: Proposed peak assignments in Raman spectra of **3** and **4** based on major peaks found in calculated spectrum of **duloxetine.H**$^{+}$ (DFT 6-31G) with frequencies scaled by 0.97.

**Description**	**duloxetine.H+** calc.	**3**	**4**
aromatic CH stretching range	3167-3130	3106-3030	3106-3018
thiophene symmetric CH stretch	3180	3096	3086
CH_3 asymmetric stretch	2905	2974	2966
CH_2 asymmetric stretch	2865	2964	2956
CH_2, CH_3 symmetric stretch	2833	2944	2930
CH stretch	2794	2902	2918
thiophene ring deformation	1633	1632	1632
naphthalene ring deformation	1605<br>1434	1582<br>1446	1576<br>1440
naphthalene ring deformation<br>CH_2 wag	1417	1434	1434
C-O stretch<br>naphthalene CH in-plane wag	1239	1240	1244
thiophene CH in-plane wag<br>CH_2 rock	1123	1146	1144
C-O stretch<br>naphthalene ring deformation	1039	1024	1022
naphthalene CH out-of-plane wag	1004	956	956
C-C str,<br>naphthalene ring deformation	913		908
naphthalene CH out-of-plane wag<br>C-C-C bend	750	746	750
naphthalene ring deformation<br>thiophene ring deformation<br>CH_2 rock	708<br>629<br>572<br>499	726<br>626<br>574<br>508	738<br>626<br>572<br>520

Table S5: Proposed peak assignments in FTIR spectra of **3** , **3.ace** and **4** based on major peaks found in calculated spectrum of **duloxetine.H**$^+$ (DFT 6-31G) with frequencies scaled by 0.97.

**Description**	**duloxetine.H+** calc.	**4**	**3**	**3.ace**
aromatic CH stretching range	3167-3130	3120-3040	3120-3040	3120-3040
aliphatic CH stretching range	2905-2794	2990-2900	2990-2900	2990-2900
C=O stretch, acetone guest	-	-	-	1710
thiophene ring deformation	1633	1634	1634	1632
naphthalene ring deformation	1570	1579	1580	1580
CH_2, CH_3 scissor	1521	1510	1507	1508
CH_2 scissor	1486	1478	1482	1480
CH_2 scissor	1475	1464	1464	1464
CH_2 rock<br>naphthalene ring deformation	1417	1442	1436	1442
C-O stretch<br>CH wag	1384	1400	1398	1398
CH_2 twist<br>CH wag	1306	1310	1320	1320
naphthalene ring deformation	1290<br>1265	-<br>-	-<br>-	-<br>-
C-O stretch<br>naphthalene ring deformation	1239	1266	1264	1266
CH, CH_2, CH_3 rock	1207	1240	1236	1236
C-C, C-N stretch	1103	-	-	-
C-O stretch<br>naphthalene ring deformation	1093	1098	1096	1096
CH_2, CH_3 twist<br>naphthalene ring deformation<br>thiophene ring deformation	1081	1070	1080	1082
C-O, C-C stretch<br>naphthalene ring deformation<br>thiophene ring deformation.	1047	1052	1066	1066
naphthalene CH deformation	1004	1021	1021	1022

CH_2, CH_3 twist				
naphthalene CH deformation<br>thiophene ring deformation	795<br>774	791<br>773	792<br>770	794<br>772
naphthalene ring deformation<br>thiophene ring deformation<br>C-C-C and C-C-C bend	757	-	-	-
thiophene CH deformation	732	733	736	734
thiophene CH deformation<br>naphthalene ring deformation<br>CH_2 rock	708	720	718	714

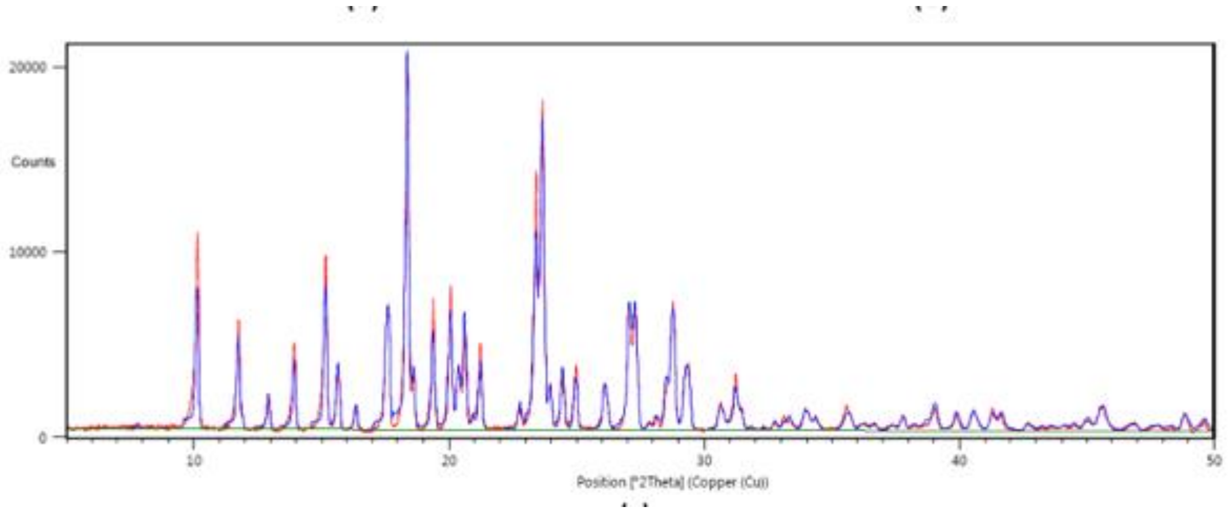

*Fig. S6: Cu K_α X-ray powder diffraction data of **4** (red) and corresponding Rietveld refinement (blue) using the synchrotron single-crystal data of **4**.*

Table S7: Subset of refined parameters from Rietveld analysis (High Score Plus, Panalytical, Netherlands) of powder XRD for **4** using the synchrotron single crystal data.

**Background**	
Method	Polynomial
Use Extended Background Terms	False
Flat Background	313.8689
Coefficient 1	1.672172
Coefficient 2	178.684
**Agreement Indices**	

Last Marquard Value	0
Condition Number	558416800
R expected	5.6136
R profile	12.60323
Weighted R profile	14.6943
D-statistics	0.10903
Weighted D-statistics	0.12988
Goodness of Fit	6.85195
**General Properties**	
Sample Geometry	Flat Plate Geometry
Automatic Cell Constraints	True
Automatic Anisotropic Displacement Constraints	True
Specimen Displacement [mm]	0.00042
Zero Shift [2Theta]	-0.03918
Polarisation Correction Coefficient	1
Linear Absorption Coefficient [1/cm]	0
Use Brindley Micro absorption Correction	False
**Refinement**	
Weighting Scheme	Against Iobs
Use summed March/Dollase Function	False
Recycle HKL's (Le-Bail Fit only)	True
Profile Function	Pseudo Voigt
Profile Base Width	4
Intensity Limit	0
Max. No. of Least-Squares Cycles	20
EPS (stop if all shifts < EPS*esd)	0.5
Job Type	X-rays
Least-Squares Type	Newton-Raphson
Marquardt Start Value	0
Max. Angle for Rietveld Asymmetry correction	60
**Relaxation Factors**	
Coordinates, isotropic Displacement and sof	0.8
Anisotropic Displacement	0.8
Scale, Zero, Background, Cell, Pref.Ori. and Bov.	0.8

FWHM, Asymmetry and Shape	0.8
**Profile Fit Settings**	
Max. No of Profile Fit Cycles	20
Profile Fit Peak Base Width	20
Use Caglioti Function	False
Use Shape Function	False
Calculate Errors	True
Asymmetry Type	Split Shape

Table S8: Torsion angle tables for molecules having alkyl chains like (a) Imipramine (b) Citalopram and (c) Fluoxetine. Angles ~180 ° indicate extended conformation, deviation from 180 ° indicate the extent of folding (highlighted in yellow).

(a)

No.	NAME	TOR1	TOR2	TOR3	TOR4
1	CAPTAZ	-128.708	-151.861	-126.744	146.303
2	COSFOF	110.302	-176.708	169.988	-176.933
3	DOHFOV	-102.561	163.679	-169.788	167.977
4	DUKTOS	134.937	179.551	166.595	162.177
4	DUKTOS	144.984	-171.483	-164.903	170.729
5	IMIPRB	137.194	-179.441	174.083	170.795
5	IMIPRB	-161.154	161.742	59.196	-171.568
6	IMIPRC	137.145	-179.655	173.5	169.388
6	IMIPRC	-159.667	160.477	60.901	-172.332
7	PROMZC01	142.876	-166.268	-151.934	163.419
8	TFPROM10	-148.476	141.643	57.789	-170.253
8	TFPROM10	138.693	-178.15	179.369	172.491
9	ROJJEG	-105.812	-178.281	176.848	-174.364
10	ROJJIK	106.113	-176.73	-170.956	166.413
11	CEJLAE	89.311	166.819	-174.716	160.263
12	IPRINC	84.293	177.003	-167.469	177.254
13	MAPTML10	80.255	-175.54	170.306	-173.482
14	CMPBIM	-96.599	173.915	164.079	-178.182
15	COSFUL	88.912	-173.476	177.647	-171.939
16	ZUWHOO	-86.28	-167.95	179.9	-168.14
17	SITYEC	-63.393	173.529	178.862	64.939
18	VICXAH	81.148	-173.25	-170.692	176.255
19	CIMPRA	-142.454	71.159	167.628	-169.961
20	GIKJOC	169.242	-59.395	-178.351	169.669
21	HAWZAG	140.319	-58.632	166.74	-178.496
22	MUWSIJ	104.408	-57.763	174.096	177.211
23	REZBII	143.414	-72.291	161.371	174.575
24	VICXAH	124.505	-60.805	-147.797	175.083
25	BOLTAX	-91.401	159.666	61.419	-169.523
26	MIGQID	-160.551	53.771	57.777	176.022
27	DUNGAV	165.178	-53.28	-59.806	-177.968
27	DUNGAV	-160.592	63.985	-168.792	-178.292
28	SINTAM10	-71.989	-179.386	-58.88	-175.396

(b)

No.	NAME	TOR1	TOR2	TOR3	TOR4
1	APFMHA	-169.176	172.211	-169.711	178.452
1	APFMHA	162.694	-172.032	170.562	-174.796
2	AZOWUI	178.378	-164.675	178.819	-65.774
3	EZIQEL	166.647	-176.338	-165.426	176.694
3	EZIQEL	170.903	176.919	171.855	122.628
4	IJUGOK	-175.27	-174.238	-163.743	-147.892
5	IQIRUX	174.202	160.44	164.692	130.87
6	SETVUJ	179.989	-174.336	-174.605	65.102
6	SETVUJ	179.989	-174.336	-174.605	-171.508
7	WASGAA	-179.403	-175.383	-175.394	-171.874
7	WASGAA	174.594	-178.973	179.51	173.61
8	WASGEE	-177.218	177.123	175.568	173.088

(c)

No.	NAME	TOR1	TOR2	TOR3
1	BPAMAL	167.078	-171.669	170.043
2	CPHMAL10	173.331	-161.183	176.873
3	DUFTOO	159.92	175.624	154.169
4	EJUKEZ	-168.786	-164.144	177.986
5	FUQTUI	-179.272	-172.62	-172.452
6	HEVDUI	-173.673	-159.098	-160.699
7	JEGWUN	168.419	-171.006	170.159
8	LUDYIV	-178.241	-168.955	171.828
9	ZITZAD	-175.682	-168.302	179.288
10	YIGNEI02	176.72	177.05	-172.94
11	ZUHFIR	-175.748	-82.045	-58.725
11	ZUHFIR	170.242	-174.634	65.094
12	FUDCOW	-175.71	-179.96	77.93
13	RAJFEO	-176.8	70.69	173.31
14	DETGIV	179.63	-157.53	-176.06
14	DETGIV	-176.84	-176.93	-61.51
15	EMIQOI	160.07	-74.97	-174.57
16	EMIQOI01	-160.03	73.27	173.88
16	EMIQOI01	166.54	-71.2	-170.44
17	EMIQOI04	159.36	-75.04	-174.97
18	RAJFAK	-169.59	69.19	174.2
18	RAJFAK	-168.17	73.62	176.1
19	S-Duloxetine	168.04	-127.34	172.84
20	S-duloxetine.ac	164.59	-78.19	178.02
21	Rac-Duloxetine	64.44	70.09	165.96

Fig. S9: Selected examples of molecular packing with molecules having an Imipramine-like side chain with (a) all four torsion angles close to 180 °, (b) either tor1 or tor2 deviating from 180 ° and (c) tor3 deviating from 180 °.

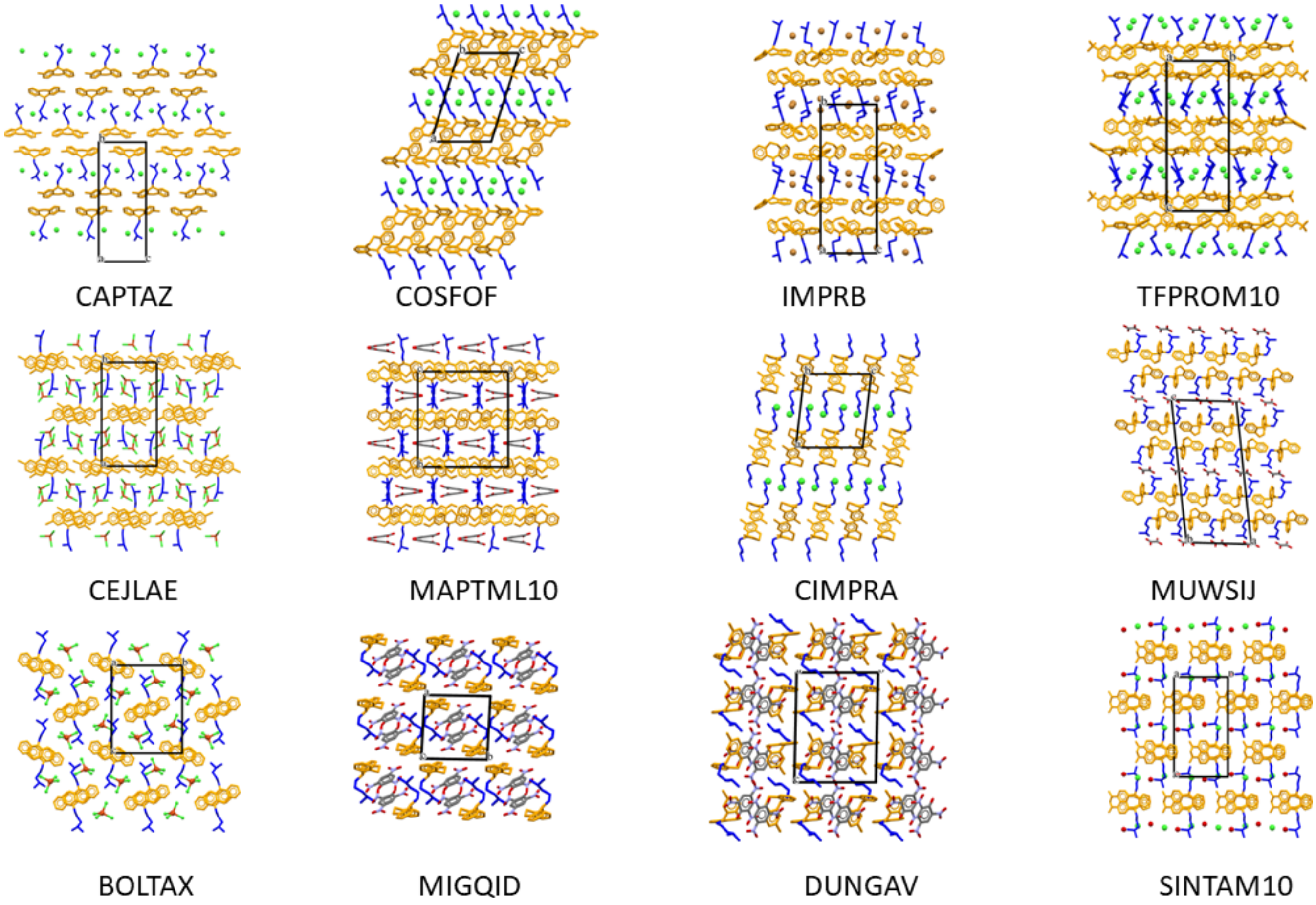

Fig S10: Examples of molecular packing in the Citalopram-like class showing all torsion angles ~180 °.

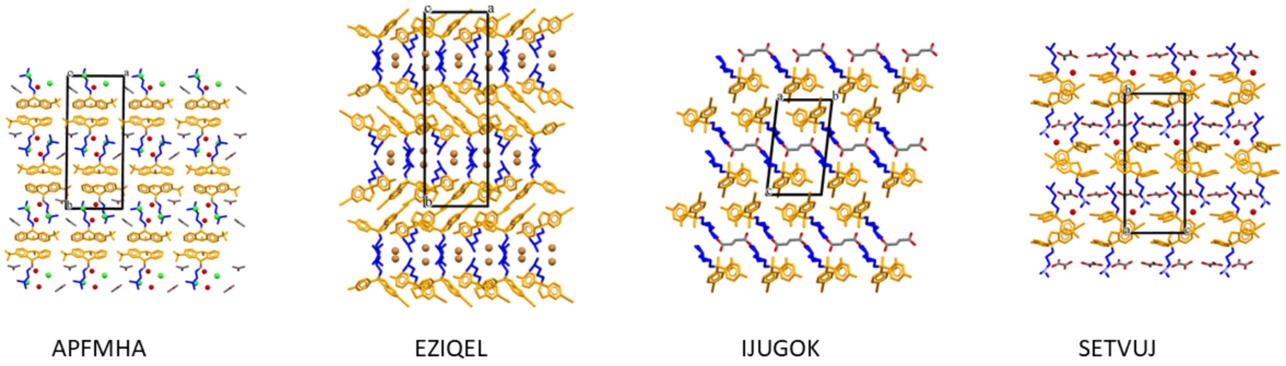

Fig. S11: Selective examples of molecules with shorter side chain similar to Fluoxetine; packing of molecules with all the three torsion angles ~180 º (a) either Tor2 or Tor3 deviating from 180 º.

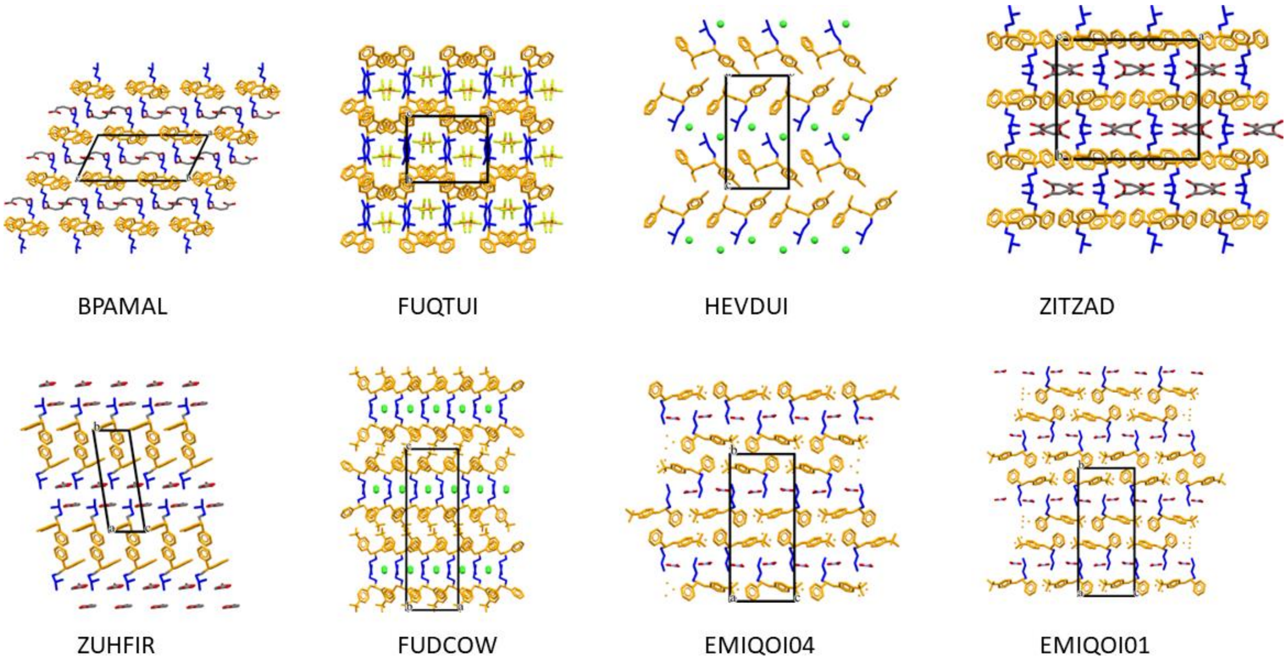

Supplement: Supplementary file 4 [file e-79-00488-sup3.pdf]
